# Atypical Familial Mediterranean Fever in a Japanese Boy with Heterozygous *MEFV* p.Ser503Cys Exon 5 Variant

**DOI:** 10.1155/2021/6650226

**Published:** 2021-03-02

**Authors:** Tomonobu Sato, Shunichiro Takezaki, Takeru Goto, Shinichi Ishikawa, Kazumi Oura, Asuka Takahata, Haruki Shiraishi, Yuji Maruo, Norio Sato, Takashi Suganuma, Makoto Mikawa

**Affiliations:** ^1^Department of Pediatrics, Kitami Red Cross Hospital, Kitami, Japan; ^2^Department of Pediatrics, Hokkaido University Hospital, Hokkaido, Japan

## Abstract

Periodic fever syndromes are heterogeneous diseases. Familial Mediterranean fever (FMF) is one of the hereditary periodic fever diseases caused by a Mediterranean fever (*MEFV*) gene abnormality. FMF can be categorized as typical or atypical, based on clinical findings and genetic screening. Atypical FMF has a wide variation of clinical findings and disease-causing mutations of *MEFV*. Therefore, it is sometimes difficult to diagnose an unknown fever as FMF. To date, a large number of various typical and atypical FMF cases have been reported in Japan. Here, we describe a Japanese boy with heterozygous *MEFV* p.Ser503Cys exon 5 variant who developed periodic fever. He was treated with colchicine; a complete eradication of his fever and various accompanying symptoms have been subsequently achieved for more than a year. Given that there have been a few reports about patients with this variant, little is known about the genetic and phenotypic role of heterozygous *MEFV* p.Ser503Cys exon 5 variant. It is therefore imperative to consider atypical FMF as a differential diagnosis when a periodic fever is encountered. Furthermore, we suggest that it is worthwhile to integrate *MEFV* gene analysis with the potential effects of colchicine treatment in patients with periodic fever.

## 1. Introduction

Periodic fever syndromes are clinically characterized by recurrent episodes of fever, systemic inflammation, and symptoms such as skin rashes, abdominal pain, chest pain, lymphadenopathy, or arthritis [1]. Familial Mediterranean fever (FMF) is one of the hereditary periodic fever diseases associated with an abnormality in the Mediterranean fever (*MEFV*) gene, which induces dysfunction of the inflammasome, followed by the production of interleukin-1*β*. In particular, FMF is caused by a dysfunction in pyrin, which is encoded by the *MEFV* gene [2]. FMF may be typical or atypical, depending on clinical findings and genetic screening [3]. The clinical characteristics of typical FMF are periodic, self-limiting fever lasting three days at most accompanied by serositis, pleuritis, peritonitis, and arthritis. FMF is considered an autosomal recessive disease, and homogeneous or compound heterozygous *MEFV* mutations in exon 10 are correlated with a typical FMF phenotype [3]. In contrast, patients with atypical FMF develop symptoms that do not necessarily match those of typical FMF and harbor variants or heterozygous *MEFV* mutations, different from those of typical FMF. In atypical FMF cases, bouts of fever, for example, last for more than four days, attacks of serositis present as localized, and nonspecific symptoms, such as arthralgia and myalgia, are observed [4]. There are no specific hematological markers for atypical FMF; therefore, diagnosis and initiation of treatment are often delayed [5]. To date, a large number of FMF variants cases have been reported in Japan [6].

Here, we report the case of a Japanese boy with heterozygous *MEFV* p.Ser503Cys exon 5 variant (NM_000243.2(*MEFV*): c.1508C > G; p.Ser503Cys) with periodic fever, improved by treatment with colchicine.

## 2. Case Presentation

**Figure 1:**
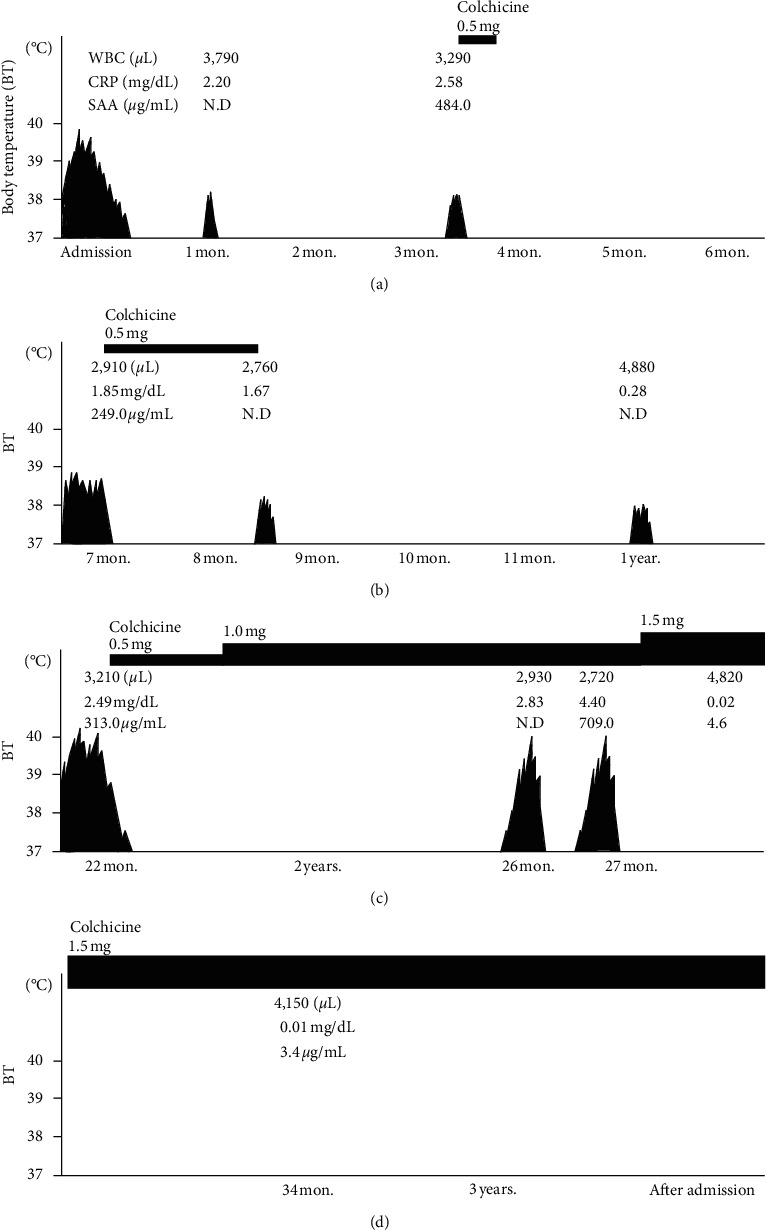
The clinical course. Horizontal axis shows months after the patient's admission, and vertical axis shows patient's body temperature (BT, notated Celsius temperature). Jagged black area means the duration and the height of patient's fever. WBC, white blood cells; CRP, C-reactive protein; SAA, serum amyloid A; N.D, no data.

An 11-year-old Japanese boy developed a prolonged fever lasting 16 days, general malaise, abdominal pain, and slight headache prior to admission. He revealed no significant chest pain or arthralgia. His medical history was unremarkable. He was diagnosed as having upper respiratory tract infection by his primary care physician and was treated with nonsteroidal anti-inflammatory drugs, antibiotics (tosufloxacin), and steroids (prednisolone, 1 mg/kg/day for 5 days). However, his prolonged fever was not eradicated, and he was referred to our department and was admitted to our ward. On admission, he was alert and oriented. He had no special family history of FMF and was not a child of a consanguineous marriage. Physical examination was unremarkable except for a body temperature of 38.4°C and aphthous stomatitis. He revealed no meningeal signs. Hematological tests were normal; blood biochemistry tests revealed the presence of high acute reactants (C-reactive protein, CRP: 3.30 mg/dL; serum amyloid A protein, SAA: 920.0 *μ*g/mL) and elevated erythrocyte sedimentation ratio (ESR, 48 mm/h). Other laboratory tests were unremarkable, including autoantibodies and viral or microbial antibody titers (Table 1). Enhanced computed tomography and a gallium scintigraphy scan revealed no abnormal findings indicating any suspicion of pleuritis or peritonitis. Bone marrow examination demonstrated no abnormal cells. No ophthalmologic disorders, such as uveitis or iridocyclitis, were detected. After screening for the cause of his fever, a follow-up was performed, and the patient received antibiotics, ceftriaxone, minocycline, and meropenem, in that order. As these drugs did not improve his symptoms, antibiotic therapy was discontinued on day 9 of admission. However, he was spontaneously afebrile subsequently. The duration of his fever was a total of 24 days. Afterwards, he was followed up as an outpatient, with the cause of the previous fever indicated as unknown. He presented periodic fever of 38-39°C that lasted for 10 days, every 2-3 months following his discharge (Figure 1). His low total Gaslini diagnostic score [7] suggested a low risk for typical FMF, tumor necrosis factor receptor-associated periodic syndrome (TRAPS), and mevalonate kinase deficiency. Due to leukocytopenia during his febrile attacks, fluorodeoxyglucose-positron emission tomography was performed with the aim of ruling out intraperitoneal histiocytic necrotizing lymphadenitis (Kikuchi–Fujimoto disease). However, we could not detect any abnormal findings, such as FDG uptake at the mesenteric lymph nodes and joints, or abnormal masses. His blood test findings including leukocyte counts and inflammatory response during the intermittent period of fever were normal.

Subsequently, he sometimes complained of knee pains and presented skin rash during the attacks. On the basis of his periodic fever (≥38°C) accompanied by elevated acute reactants such as CRP and SAA, we suspected an autoinflammatory syndrome and treated him with colchicine (0.5 mg/day, 0.015 mg/kg/day). The patient was afebrile a day after colchicine administration. A gene analysis, including *MEFV*, *TNFRSF1A*, *NLRP3*, *MVK*, *PLCG2*, *NLRC4*, *ADA2*, *TNFAIP3*, *TREX1*, *RNASEH2A*, *RNASEH2B*, *RNASEH2C*, *SAMHD1*, *ADAR*, and *IFIH1*, showed the presence of a heterozygous *MEFV* p.Ser503Cys exon 5 variant. Although the long febrile duration and leukocytopenia during the attacks were not typical symptoms of FMF, the presence of abdominal pain, the efficacy of treatment with colchicine, and the detection of heterozygous *MEFV* p.Ser503Cys exon 5 variant suggested that he had an atypical FMF. He was initially treated with colchicine only during the febrile attacks. As complete elimination of the attacks was not gained, he was treated with colchicine regularly. Although a daily administration of 0.5 mg (0.01 mg/kg/day) colchicine did not prevent his febrile attacks, a daily dose of 1.5 mg (0.03 mg/kg/day) colchicine achieved complete eradication of the attacks for more than one year.

## 3. Discussion

In this study, we described a Japanese boy with heterozygous *MEFV* p.Ser503Cys exon 5 variant with periodic fever, which was improved by administration of colchicine. *MEFV* heterozygous p.Ser503Cys exon 5 variant is reportedly found in approximately 2% of the Japanese population and in 0.6%–1.9% of Japanese patients with FMF, a frequency higher than that observed in other races [3, 6, 8]. Moreover, FMF usually develops in patients with homozygous or compound heterozygous mutations in the *MEFV* gene. Therefore, it is possible that the heterozygous *MEFV* p.Ser503Cys exon 5 variant is one of the single nucleotide polymorphisms occurring in the Japanese population, and the patient's manifestations may have been incidental. However, in Japan, there are more patients with FMF, carrying heterozygous mutations in the *MEFV* gene than those carrying homozygous mutations [3]. In addition, in a limited number of cases with a heterozygous *MEFV* p.Ser503Cys exon 5 variant, the patients have been phenotypically diagnosed with atypical FMF (Table 2) [9–11]. It remains perplexing why patients with heterozygous *MEFV* p.Ser503Cys exon 5 variant present atypical FMF manifestations. Some reports indicate that other factors, such as polymorphisms of human leukocyte antigen (HLA) or polymorphism in the gene associated with serum amyloid A, influence the risk of FMF expression [12, 13]. Moreover, although we did not detect any additional gene mutations, a previous report describes a patient with a mutation in the NOD-like receptors family pyrin domain-containing 3 (*NLRP3*) gene, which is one of the molecules constituting the inflammasome, in addition with the heterozygous *MEFV* p.Ser503Cys variant [11]. Additional mutations in genes involved in inflammation might be required for the development of symptoms in patients with heterozygous *MEFV* p.Ser503Cys variant. However, a review of a greater number of heterozygous *MEFV* p.Ser503Cys exon 5 variant cases is essential to speculate about the underlying pathophysiological mechanisms of atypical FMF and establish the best approach for its management.

In our case, the patient developed periodic fever after age 10 and demonstrated fever, accompanied with aphthous stomatitis and slight abdominal pain. Although blood biochemistry tests revealed the presence of high acute phase reactants and elevated erythrocyte sedimentation ratio, his complete blood counts often revealed leukocytopenia during the fever bouts. Moreover, treatment with increasing doses of colchicine improved his periodic fever and accompanying symptoms. Although the detailed pathophysiology is unknown, previous reports describe that similar to our case, atypical FMF patients demonstrated leukopenia during febrile attacks [14, 15]. However, these patients were not reported to have the *MEFV* exon5 p.Ser503Cys variant, indicating that leukocytopenia during febrile attack in atypical FMF is not limited to the *MEFV* exon5 p.Ser503Cys variant.

According to the Gaslini diagnostic score, the presence of aphthous stomatitis during febrile episodes and the high age of onset are the factors that do not positively indicate typical FMF, TRAPS, or mevalonate kinase deficiency [7]. The presence of aphthous stomatitis especially made it difficult to distinguish the disease from other autoinflammatory and autoimmune diseases, such as PFAPA syndrome and Behcet's disease (BD). Without disease-specific tests such as colonoscopy and/or HLA analysis, it is hard to rule out BD, and there is a possibility that the patient was in an early stage of BD. However, in our case, though aphthous stomatitis was present, the skin, ocular, and vulvar symptoms, which are characteristic of BD, were not observed. In addition, no vascular or central nervous system lesions were found, and laboratory tests revealed no leukocytosis. Hence, we concluded that it was unlikely that this patient had BD. Recently, there have been reports on a group of diseases exhibiting a BD-like phenotype. Perazzio et al. reported that a BD-like phenotype is at the crossroads of autoimmune and autoinflammatory syndromes and is associated with a variety of congenital immune disorders including FMF [16]. Since BD and FMF are overlapping disease groups, it could be difficult to make a strict distinction between them.

However, the presence of abdominal pain, which is suspicious of nonlocalized peritonitis, and the efficacy of colchicine in the reduction of fever were factors that suggested atypical FMF. To diagnose FMF, either the Tel Hashomer criteria or the criteria of the diagnostic tool for FMF, proposed by the Ministry of Health, Japan, are referred [17–19]. According to Tel Hashomer diagnostic criteria, in this case, his symptoms were consistent with fever as a major criterion and the effectiveness of colchicine as a minor criterion. However, he could not be regarded as a typical FMF patient as he had febrile episodes lasting longer than three days and no accompanying symptoms such as pleuritis or arthritis. Notably, in Japan, the number of patients with atypical FMF exceeds those with typical FMF with *MEFV* exon 10 mutation, with symptoms such as peritonitis and pleuritis, which are common in typical FMF patients of other ethnic groups. Therefore, in addition to Tel Hashomer criteria, the diagnostic criteria for FMF proposed by the Ministry of Health, Japan, are also used [19], which propose that fever with an elevation of serum acute phase reactants is considered an essential criterion, and the presence of symptoms due to serositis such as pleuritis or the effectiveness of colchicine are considered auxiliary criteria for a clinical diagnosis of FMF. In addition, patients with *MEFV* exon 10 mutations are diagnosed with typical FMF, and patients with mutations in other regions of the *MEFV* gene are diagnosed with atypical FMF. In the present case, the patient demonstrated prolonged fever for more than four days, accompanied by elevated serum acute phase reactants, abdominal pain, headache during febrile episodes, and fever remission upon treatment with colchicine. The *MEFV* variant amino acid substitution and his clinical manifestations suggest the patient's diagnosis as an atypical FMF according to diagnostic criteria prescribed by the Ministry of Health, Japan.

Our patient had frequent complaints of headache, which is not included in the symptoms of FMF. We did not perform further evaluation of his cerebrospinal fluid; however, a study reported an atypical FMF patient with compound heterozygous mutations in exons 2 and 5 (E148Q/S503C) in *MEFV* who experienced recurrent headache due to chronic aseptic meningitis [9]. Although it is difficult to strongly suggest from such a small number of cases, it is possible that headache due to aseptic meningitis is one of the complications in atypical FMF, and atypical FMF could be a differential diagnosis in periodic fever patients with headaches.

In conclusion, here, we reported a Japanese boy with heterozygous *MEFV* p.Ser503Cys exon 5 variant with periodic fever that was improved by treatment with colchicine and remains afebrile to date. Since there have been few reports on patients with this variant, little is known about its genetic and phenotypic role. When such cases are encountered, therefore, a consideration should be given for an atypical FMF differential diagnosis. Furthermore, for these cases, we speculate that it is worth conducting an *MEFV* gene analysis and testing colchicine treatment for potential relief. Early diagnosis and therapeutic intervention for suspected FMF patients is pivotal to avoid the accumulation of SAA and especially to understand the clinical characteristics and therapeutic strategies for suspected patients of atypical FMF with heterozygous *MEFV* p.Ser503Cys exon 5 variant.

## Figures and Tables

**Table 1:** Laboratory findings on admission.

WBC	5,460/*μ*L	Na	137 mEq/L		Autoantibodies	Neg
NEU	78%	K	4.0 mEq/L			
LYM	15%	Cl	98 mEq/L		*M. pneumoniae* -IgM	Neg
MONO	7%	Ca	9.1 mg/dL		EBV VCA-IgG	<10 1:n
RBC	498 × 10^4^/*μ*L	IP	4.6 mg/dL		EBV VCA-IgM	<10 1:n
Hb	13.7 g/dL	CRP	3.30 mg/dL		EBNA	160 1:n
Ht	40.9%	Procalcitonin	0.58 ng/mL	(<0.50)	CMV-IgG	Neg
PLT	27.9 × 10^4^/*μ*L	ESR (1 h)	43 mm		CMV-IgM	Neg
RET	1.2%	IgG	1,270 mg/dL		*Coxiella burnetii-*IgG (paired serum)	<64 1:n
AST	31 IU/L	IgA	228 mg/dL			
ALT	25 IU/L	IgM	120 mg/dL			
*γ*-GTP	14 IU/L	Ferritin	57 ng/mL	(21–282)	Urinalysis	
LDH	231 IU/L	sIL-2R	498 U/mL	(122–496)	pH	6.5
CK	23 IU/L	ANA	<40 1:n		Red blood cell	Neg
T-BIL	0.3 mg/dL	CH50	64 U/mL		White blood cell	Neg
TP	7.5 g/dL	C3	205 mg/dL		Protein	Neg
Alb	4.0 g/dL	C4	51.7 mg/dL			
BUN	20.0 mg/dL	RF	<15 U/mL			
CRE	0.83 mg/dL	MMP-3	30.1 ng/dL	(37–121)		
		SAA	920.0 *μ*g/mL	(<8.0)		
		*Β-*2 microglobulin	2.8 mg/L	(0.0–2.0)		

sIL-2R, soluble interleukin-2 receptor; ANA, antinuclear antibody; RF, rheumatoid factor; MMP-3, matrix metalloproteinase-3; SAA, serum amyloid A; *M. pneumoniae*, *Mycoplasma pneumoniae*; EBV, Epstein–Barr virus; VCA, virus capsid antigen; EBNA, EBV nuclear antigen; CMV, cytomegalovirus; Neg, negative; (), normal range.

**Table 2:** Profile of previously reported FMF patients with *MEFV* p.Ser503Cys exon 5 variants.

Patient (Ref.)	Age (yr)/sex	*MEFV*	FMF	Febrile attack		Other symptoms of FMF				
				Frequency (mo)	Duration (day)	Ab.	Th.	Arth.	Head.	Response to colchicine
[9]	38/male	E148Q/S503C	Atyp.	<1	14	+	+	−	+	+
[10]	47/male	S503C/—	Atyp.	2	3	+	−	+	+	+
[11]	65/male	S503C/—	Atyp.	NA	NA	+	−	+	NA	+
Our case	11/male	S503C/—	Atyp.	1–3	10	+	−	−	+	+

Ab., abdominal pain; Arth., arthralgia; Atyp., atypical FMF; FMF, familial Mediterranean fever; Head., headache; mo, month; NA, not available; Ref.; reference number; Th., thoracic pain; Typ., typical FMF; yr, year.
